# Aging and wisdom: age-related changes in economic and social decision making

**DOI:** 10.3389/fnagi.2015.00120

**Published:** 2015-06-18

**Authors:** Kenneth Teck Kiat Lim, Rongjun Yu

**Affiliations:** ^1^Department of Psychology, National University of SingaporeSingapore, Singapore; ^2^Neurobiology/Aging Programme, Life Sciences Institute, National University of SingaporeSingapore, Singapore; ^3^Center for Life Sciences, Singapore Institute for Neurotechnology (SINAPSE), National University of SingaporeSingapore, Singapore

**Keywords:** aging, wisdom, decision making, social conflicts, emotion

## Abstract

World life expectancy is increasing and many populations will begin to age rapidly. The impeding prevalence of a greater number of older people living longer lives will have significant social and economic implications. It is important to understand how older people make economic and social decisions. Aging can be associated with a “phenomenon of decline” and also greater wisdom. This paper seeks to examine the relationship between wisdom and aging. It reviews and connects the behavioral sciences and neuroscience literature on age differences in the following social and economic decision making domains that represent subcomponents of wisdom: (1) prosocial behavior in experimental economic games and competitive situations; (2) resolving social conflicts; (3) emotional homeostasis; (4) self-reflection; (5) dealing effectively with uncertainty in the domains of risk, ambiguity and intertemporal choice. Overall, we find a lack of research into how older people make economic and social decisions. There is, however, some evidence that older adults outperform young adults on certain subcomponents of wisdom, but the exact relationship between old age and each subcomponent remains unclear. A better understanding of these relationships holds the potential to alleviate a wide range of mental health problems, and has broad implications for social policies aimed at the elderly.

## Introduction

“With age comes wisdom, but sometimes age comes alone.”

*—Oscar Wilde*

World life expectancy has been steadily rising in the past two centuries and is expected to continue increasing for the foreseeable future (Oeppen and Vaupel, 2002). Increased longevity is especially pronounced in developed countries compared to developing ones (Mathers et al., 2001). Based on a steady increase of almost 3 months of life per year, it may not be uncommon for those born since 2000 in certain developed countries, such as USA, the UK, Japan and other western European countries, to live for 100 years (Christensen et al., 2009; Vaupel, 2010). This is astonishing given that the average life expectancy in developed countries was under 45 years in 1900 (Juvin, 2010). The population is also expected to continue to age rapidly over the next few decades (Lutz et al., 2008). There are serious economic and social implications of our increasing longevity and rapidly aging population (Schneider and Guralnik, 1990; Lloyd-Sherlock, 2000; Tinker, 2002; Poterba, 2004; Bloom et al., 2010b; see Bloom et al., 2010a). The extra years of lives will need to be financed and there will be less people in the labor force than outside of it based on current retirement age laws (Sierra et al., 2009). The roles of social norms surrounding education, employment and retirement will need to be rethought so that the elderly can continue to contribute without impinging on the prospects of the younger citizens (Vaupel and Gowan, 1986; Vaupel and Loichinger, 2006).

Traditionally, research on aging has focused on the cognitive aspects of age-related changes. Old age is associated with declines in many aspects of cognition (reviewed in Hedden and Gabrieli, 2004; Raz and Rodrigue, 2006; Park and Reuter-Lorenz, 2009), as well as with a variety of detrimental stereotypes of incompetence (see Cuddy et al., 2005; Kite et al., 2005; North and Fiske, 2012). Recent major theories on aging, however, emphasize that emotion and motivation play a fundamental role in shaping age related changes in decision making and well-being. The Socioemotional Selectivity Theory (Carstensen et al., 1999) proposes that time horizons influence goals and people engage in a lifelong selection process of strategically and adaptively cultivating their social networks to maximize social and emotional gains and minimize social and emotional risks. When time is perceived as open-ended, goals are most likely to be preparatory and to be used to optimize the future, e.g., gathering information, experiencing novelty and expanding breadth of knowledge. As a result, young adults may place the greatest emphasis on the potential for information gain and future contact. When constraints on time are perceived, goals focus more on objectives that can be realized in their pursuit to maximize meaningful activities in the present. As a consequence, goals emphasize feeling states, particularly regulating emotional states to optimize well-being. Thus, elderly people tend to place the greatest emphasis on the potential for affective gain. Strength and Vulnerability Integration Theory (Charles, 2010) incorporates the socioemotional selectivity theory and further states that aging is associated with strengths in emotion regulation that entail the use of attentional, appraisal, and behavioral strategies of emotion, as well as vulnerabilities in emotion regulation as a consequence of reduced physiological flexibility, especially in situations that elicit high levels of sustained emotional arousal. The Motivational Theory of Life-Span Development (Heckhausen et al., 2010) proposes that individuals in late adulthood shift from primary control processes that are directed at changing the world by bringing the environment in line with one’s wishes to secondary control processes aimed at changing the self to bring oneself in line with environmental forces. To meet the major challenges faced in old age, individuals need to increasingly resort to secondary control strategies of adjusting expectations, values, and attributions in order to pursue more attainable goals when certain primary control goals become unattainable. These theories suggest that aging is associated with dramatic changes in personal goals and highlight the strategies and skills used to achieve these new goals.

While aging is generally viewed as a “phenomenon of decline”, there is an aspect to it that “holds more promise than present reality may reveal”: wisdom (Baltes and Staudinger, 1993). The concept of wisdom has its roots in religion and philosophy (see Ardelt, 2004; Baltes and Smith, 2008). Wisdom is a complex, multi-faceted construct and there is no consensus on its definition, instead there are a variety, of mostly overlapping, theories of wisdom (Baltes and Staudinger, 2000). This has been largely encapsulated and distilled into six subcomponents by Meeks and Jeste (2009): (1) prosocial attitudes/behaviors; (2) social decision making/pragmatic knowledge of life; (3) emotional homeostasis; (4) reflection/self-understanding; (5) value relativism/tolerance; and (6) acknowledgment of and dealing effectively with uncertainty.

Despite empirical research into the construct of wisdom spanning more than three decades, the topic of wisdom continues to be overlooked by the neuroscience and psychology communities (Jeste and Harris, 2010) and only has gained attention in recent years in the field of aging-related research. This paper will review and connect the behavioral sciences and neuroscience literature on wisdom and aging, and will be organized around the theoretical framework of Meeks and Jeste (2009). For this review, we integrate the two subcomponents of (2) social decision making/pragmatic knowledge of life, i.e., knowing how to successfully navigate challenging social situations, and (5) value relativism, i.e., tolerance of another person’s or culture’s value systems, into one: “resolving social conflicts”. Our rationale is that both of these subcomponents of wisdom are essential to making sound social decisions. We begin with a review of the literature on age differences in prosocial behaviors, specifically on experimental economic games.

## Prosocial Behaviors

Wisdom entails the ability to achieve a common social good. Prosocial behavior broadly refers to acting beyond one’s self-interest to benefit other people in one’s social group and/or society (Penner et al., 2005). In economics, there are three major types of prosocial behavior: reciprocity, inequity aversion and altruism (see Fehr and Fischbacher, 2002). They are usually measured by the amount of a finite amount of money that is split with another person in experimental economic games. Reciprocity refers to responding in a similar manner to the actions of another person. This depends on the perception of (un)fairness of the actions of the other person, which is determined by whether split amounts are deemed equitable. Inequity aversion refers to wanting to achieve an equitable split of outcomes. This includes wanting to increase or decrease the amount allocated to another person who falls short or exceeds the equitable threshold respectively. Altruism refers to always, i.e., unconditionally, responding positively to another person’s action. This means never wanting to decrease another person’s allocated amount.

**Figure 1:**
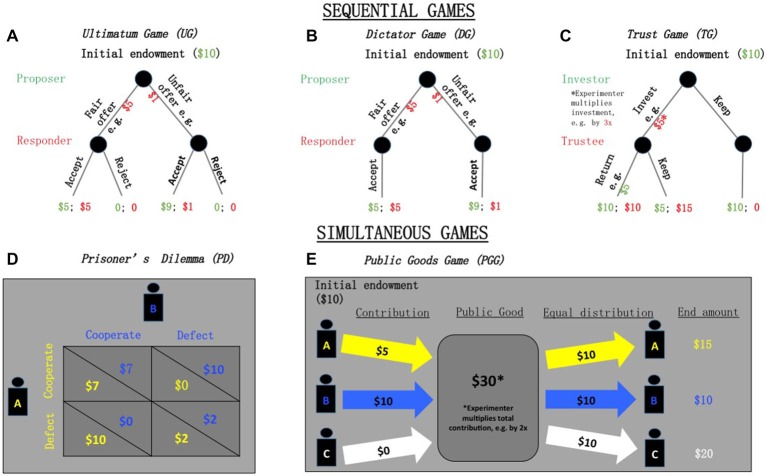
**The behavioral game paradigms**. In sequential games, such as the ultimatum game (UG) **(A)**, dictator game **(B)**, and trust game **(C)**, games are played sequentially, i.e., players move one at a time and the move of a preceding player is known. In simultaneous games, such as prisoner’s game **(D)**, and public goods game **(E)**, players make their moves at the same time.

Standard economic theory assumes that “*all* people are *exclusively* motivated by their material self-interest” and thus, do not care about the well-being of others (Fehr and Schmidt, 1999). That is, players will maximize their self-interest at the expense of others—even in experimental games. However, an overwhelming number of studies have consistently rejected this standard economic “self-interest hypothesis” and have provided evidence showing that individuals also have prosocial motivation (for reviews, see: Fehr and Schmidt, 2001; Meier, 2006; Levitt and List, 2007; Henrich et al., 2010). There are at least five common economic games to measure prosocial behavior: ultimatum, dictator, trust, prisoner’s dilemma and public goods. These experimental games are traditionally played under complete anonymity (to the other player and the experimenter). Depending on the type of prosocial behavior of interest, these games may be played either one-shot, i.e., once, or repeated. These games differ in whether they are played simultaneously, i.e., players make their moves at the same time, or sequentially, i.e., players move one at a time and the move of a preceding player is known. In the next section, we will review older people’s prosocial behavior on these games. Although there are numerous studies examining prosocial behavior on these games, only a handful have examined its relationship with old age. We will begin with a review of ultimatum game (UG)s (see Figure 1A).

### Ultimatum Game

The classic UG is a two-player, sequential game that involves splitting a sum of money between a Proposer and a Responder (Güth et al., 1982). The Proposer is endowed with a fixed amount of money, say, $10, and has to propose some amount, *x*, to another person, whose identity is not known. The Responder has two choices: accept or reject the proposed offer. If the offer is accepted, the Responder gets *x*, while the Proposer gets $10-*x*; if the offer is rejected, both players receive nothing (See Thaler, 1988, for a readable description of the common UG variations). The standard economic “self-interest hypothesis” predicts that the Proposer will offer the minimum amount possible (e.g., one cent), which would be accepted by the Responder who values this over nothing. However, this is very rarely the case (see Güth and Tietz, 1990; Güth, 1995; Cooper and Dutcher, 2011; Güth and Kocher, 2014). A meta-analysis of 37 papers found that, on average, Proposers offered 40% of their “pie”, i.e., endowment, to Responders (Oosterbeek et al., 2004). Offers for larger pie sizes and shares tend to be rejected less often (Oosterbeek et al., 2004); many studies report that it is not uncommon for offers of less than 40–50% of the pie to be rejected (see Cooper and Dutcher, 2011). This willingness to punish unfair behavior while incurring a cost reflects the degree of social inequity aversion.

We report a total of five studies that examine the prosocial behavior of older people on UGs. Two studies examined age differences for Proposers and reported different results. One study found that older participants proposed more generous offers than younger participants in the standard UG (Bailey et al., 2013), while another found no age differences (Beadle et al., 2012). Studies examined age differences for Responders’ acceptance/rejection rates with mixed results. Two studies did not find any age differences (Beadle et al., 2012; Bailey et al., 2013). One study reported no difference for fair (50% of the pie) and unfair (10% and 20% of the pie) offers but older participants were more likely to reject moderately unfair (30% of the pie) offers (Harlé and Sanfey, 2012). It was found that older participants rejected more unfair offers (Roalf et al., 2012). When the unfairness was self-advantageous, older participants were more likely to reject very high offers, which suggests inequity aversion since they do not want to be unfair to the other party (Bellemare et al., 2011).

Overall, older participants exhibit at least equal, or perhaps even more, prosocial behavior as Proposers. As Responders, older people appear to display greater inequity aversion, even in self-advantageous conditions.

### Dictator Game

The classic dictator game (DG; see Figure 1B) is similar to the UG, except that the Responder cannot reject, and must accept, the amount offered by the Proposer, i.e., the “dictator” (Kahneman et al., 1986; Forsythe et al., 1994). The standard economic “self-interest hypothesis” predicts that the Proposer will offer nothing. However, a meta-analysis of 129 experiments reported that 63.89% of participants offered a positive amount with an average of 42.64% of the pie, while 36.11% offered nothing (Engel, 2011). Further, age had “a strong effect”: giving nothing decreased with age and “never happens in the elderly”, who give at least 50% of the pie almost all of the time (Engel, 2011). It is also worth reporting the results of three other studies that specifically examined the relationship between prosocial behavior on DG and older people. These studies were published after the meta-analysis. Two studies did not find any significant relationship (Roalf et al., 2012; Rieger and Mata, 2015). The other study used a modified DG by inducing empathy into dictators and found older people to give significantly more (Beadle et al., 2013).

Overall, older people seem to exhibit more prosocial behavior in the DG. It is worth highlighting a surprising finding from the meta-analysis: the mode for elderly contribution was 100% of the pie (Engel, 2011). However, more studies are needed to investigate age differences in the DG since 94.7% of dictators in the meta-analysis were students and only 0.7% was elderly people.

### Trust Game

The classic trust game (TG; see Figure 1C) is a two-player, sequential game that involves splitting a sum of endowed money between an Investor and Trustee in two stages (Berg et al., 1995). In the first stage, both players are endowed with a fixed amount of money, say, $10, which the Investor can choose to invest or keep. If the Investor chooses to keep the money, the game ends. If the Investor chooses to invest, then an amount, *x*, must be specified. This amount, *x*, is then tripled by the experimenter so that the Trustee receives 3*x* In the second stage, the Trustee can decide whether to return the money to the Investor. If the Trustee decides to keep the money then the game ends with the Investor having a total of $10−*x* and the Trustee, $10 + 3*x* If the Trustee chooses to return some money, *y*, then the game ends with the Trustee earning a total of $10 + 3*x* −*y* and the Investor, $10−*x*+*y*. However, in many replications of game, the Trustee is not endowed with any money at the beginning (Johnson and Mislin, 2011).

The TG can be viewed as a variant of the DG, where the Trustee dictates an amount that was initially allocated by the Investor (Camerer and Fehr, 2004). The standard economic “self-interest hypothesis” predicts that no money will be invested since the Investor will anticipate the Trustee keeping all of the investment and not returning anything. This hypothesis is, however, not supported by empirical findings. A meta-analysis of 162 experiments involving more than 23,000 participants found Investors to invest 50% of the endowment on average, and Trustees to return 37% of the total amount they had available (Johnson and Mislin, 2011).

Individual studies examining the relationship of older people’s prosocial behavior on TGs report mixed results. Three studies found no significant relationship for amount invested and amount returned by Trustees (Etang et al., 2011; Johansson-Stenman et al., 2013; Rieger and Mata, 2015). One study found older people to invest and return more (Sutter and Kocher, 2007). Three studies found older people to invest less, of which two found older people to return more (Fehr et al., 2003; Bellemare and Kröger, 2007), while the other did not measure this relationship (Holm and Nystedt, 2005). The meta-analysis did not examine age but found students to return less money compared to non-students and no difference for amount invested (Johnson and Mislin, 2011), which could suggest that older people invest more since non-students are generally older than students.

Overall, there is no clear evidence on the prosocial behavior of older people playing TGs based on the seven studies reviewed. Of the seven studies that examined Investor behavior, three found no significant relationship with age, three found a negative relationship and one found a positive relationship. Six of the seven studies examined Trustee behavior and half found no significant relationship with age while the other half found a positive relationship.

### Prisoner’s Dilemma

The classic prisoner’s dilemma (PD; see Figure 1D) is a two-player, simultaneous game where each player can either choose to cooperate or defect. How much each player earns depends jointly on the choices made by both players. There are three possible scenarios with their respective payoffs in parentheses, where a larger number represents a higher payoff: one player cooperates while the other defects (1:4), both players cooperate (3:3), and both players defect (2:2).

The PD tests whether players reciprocate expected cooperation (Camerer and Fehr, 2004). The standard economic “self-interest hypothesis” predicts that each player will choose to defect, which would lead to both players earning the second lowest payoff. Although mutual cooperation would lead to the second best outcome, there is the possibility of earning the worst payoff if the other player defects. This is also known as the Nash equilibrium: both players can do no better in terms of payoffs than to defect (Nash, 1951). However, there is overwhelming evidence of cooperation in experimental PD games (Dawes, 1980; Sally, 1995; Cooper et al., 1996; Brosig, 2002; Jones, 2008; Balliet, 2009).

Although the PD is one of the most well-known games, there are no studies examining the performance of older people. We identified 124 unique PD studies from four meta-analysis studies (Balliet, 2009; Balliet et al., 2009, 2011a,b). Of the 119 studies that we could access, 108 used student samples (90.7%). The remaining 11 studies either did not report age, or had a mean age of <37 years old. More studies involving older participants are required for a better understanding of their performance on PD.

### Public Goods Game

The classic public goods game (PGG; see Figure 1E) is a generalized form of PD involving multiple players. Players are endowed with some amount of money, which they can choose whether to contribute to a “public good” or not. If players contribute (cooperate), the sum of contributions are multiplied by some factor, *m*, and distributed evenly to all players, including those who did not contribute. If players do not contribute (defect), they keep their money and stand to gain from the distribution based on the contribution of other players.

While the group would benefit most if everyone contributed, the standard economic “self-interest” hypothesis predicts no contribution at all. This hypothesis, is however, not supported by empirical findings (Ledyard, 1995; Chaudhuri, 2011). A meta-analysis of 27 studies involving 711 groups of participants found the average contribution to be 37.7% of the total endowment (Zelmer, 2003). Only one study examined the relationship between older people’s prosocial behavior in a PGG and it reported a concave result, i.e., the middle-aged participants contributed the most compared to older and younger participants (Rieger and Mata, 2015). More studies involving older participants are required for a better understanding of their performance on PGG.

### Competitive Behavior

The willingness and ability to compete is usually important for the economy. It has been suggested that prosocial tendencies increase while competitive behavior declines with older age (see Mayr et al., 2012). There are three behavioral experiments that examine competitive behavior in old age (Charness and Villeval, 2009; Mayr et al., 2012; Sproten and Schwieren, 2015). In these experiments, participants are given a task, e.g., simple mental arithmetic, and the choice of payment based on absolute or relative performance. Choosing to be paid based on performance relative to other participants and doing well reflects a willingness and ability to compete respectively. Two of the three studies found no significant age differences in willingness to compete (Charness and Villeval, 2009; Sproten and Schwieren, 2015), while the other found an inverted U-shaped relationship: willingness increased up to 50 years old and declined after Mayr et al. (2012). All three studies report no significant age differences in ability to compete. Taken together, these findings mostly suggest that older people remain willing and able to compete.

### Summary

We surveyed the literature on the prosocial behavior of elderly people across five common types of experimental games. Results were mixed and there was no clear evidence of age differences in prosocial behavior across these games. We also identified several important issues consistent across games that need to be addressed in the future. First, there are only a few studies involving older participants. For some games such as PD, there were no studies examining age differences. Second, most of these studies involve small samples, e.g., 18 young adults vs. 20 older adults (Harlé and Sanfey, 2012). Third, there were many sources of heterogeneity across studies. There were differences in whether studies employed a between- or within-subjects design and controlled for confounding factors such as income, as well as the average age of participants and in the design of the games, e.g., one-shot vs. repeated, amount endowed, etc. We also surveyed the literature examining the competitive behavior of older people in experiments where participants can choose to be paid based on absolute or relative performance. Overall results from three studies suggest that older people generally remain willing and able to compete.

## Resolving Social Conflicts

The ability to resolve social conflicts can be viewed as possessing social wisdom and refers to recognizing and respecting differences in individuals’ value systems and employing pragmatic reasoning to successfully navigate social issues in life with a preference for compromise (Basseches, 1980; Kramer, 1990; Baltes and Smith, 2008). There is empirical evidence that social wisdom improves with age. Older people tend to use more complex reasoning schemas that emphasize multiple perspectives and compromise when faced with various social dilemma scenarios (Grossmann et al., 2010). However, gains in social wisdom may be influenced by cultural differences in the socialization of interpersonal harmony and conflict avoidance. For example, there were age differences in social wisdom between Japanese and American adults, but this depended on whether the dilemma was interpersonal or intergroup (Grossmann et al., 2012). Further, contrary to the adage “with age comes wisdom, a recent study found no age differences in wise reasoning about personal conflicts in American adults (Grossmann and Kross, 2014). Moreover, both younger and older adults exhibited similar amounts of the self-distancing effect, i.e., reasoning more wisely about other people’s social problems than about their own. The lack of age-related difference in wise reasoning was also observed in another study, which asked participants about nonthreatening, but still rather age-neutral area of the self, e.g., “Please think aloud about yourself as a friend” (Mickler and Staudinger, 2008). These findings suggest that social wisdom may not be a universal and homogenous construct, and highlight the need for more studies on samples from different countries and cultures.

Everyday problem-solving/decision-making effectiveness (EPSE) is another domain that involves resolving social conflicts. EPSE incorporates both real world decision making and everyday problem solving abilities (see Thornton and Dumke, 2005). EPSE tasks typically assess the number of “safe and effective” solutions participants can generate to everyday social problems, e.g., “What should an elderly woman who has no other source of income do if her social security check does not come in 1 month?” (Heidrich and Denney, 1994). A higher number of solutions generated reflects greater EPSE. A meta-analysis of 28 studies (*N* = 4482) found an overall decline in EPSE among older participants (Thornton and Dumke, 2005). Moderator analyses revealed that age differences in EPSE were substantially reduced when problems were interpersonal and when older adults were highly educated (Thornton and Dumke, 2005). We also found that most, if not all, of the 28 studies were conducted in Anglo countries.

## Emotional Homeostasis

Successful emotion regulation that maintains emotional homeostasis is crucial to wisdom. Old age is generally perceived as “Doom and gloom”, which characterizes later life as a time of profound physical, cognitive, and emotional declines. Yet recent empirical and theoretical work challenges this view by illustrating the “bright side” of aging. Accumulating evidence shows that older adults attend to and remember positive vs. negative information to a greater extent than younger adults (Mather and Carstensen, 2005). A recent meta-analysis of age-related positivity effect confirmed that older adults show a significant information processing bias toward positive, but not negative, information (Reed et al., 2014). In corroborating with these behavioral evidence, recent neuroimaging studies found a relative reduction in activation during loss anticipation paralleled by significantly weaker negative arousal for large loss cues in older adults, despite intact striatal and insular activation during gain anticipation with age (Samanez-Larkin et al., 2007). Healthy older adults also exhibited enhanced activity in the nucleus accumbens in response to an expected reward value (Chowdhury et al., 2013). A recent neuroimaging study demonstrated that responsiveness to regret was specifically reduced in successful aging paralleled by autonomic and frontostriatal characteristics indicating adaptive shifts in emotion regulation, suggesting that disengagement from regret reflects a critical resilience factor for emotional health in older age (Brassen et al., 2012). Taken together, recent research indicates that older adults may outperform younger adults in maintaining emotional homeostasis, which might contribute to their wisdom in dealing with life challenges.

## Self-Reflection

The concept of self is very complex and includes various types of self-directed internal thought processes including autobiographical reminiscence, self-referencing, self-esteem, and so on. The ability to reflect on self is an essential prerequisite for insight. To date, the vast majority of studies examining age-related changes in self-understanding have focused on self-referential processes. Self-referential processing takes place when an individual encodes information into memory in reference to the self (Rogers et al., 1977; Symons and Johnson, 1997). Recent behavioral studies found that self- and close other-referencing similarly enhance memory for both young and older adults relative to the distant other people condition, suggesting that self-referencing provides an age-equivalent boost in memory (Hamami et al., 2011). Other studies found that elderly subjects were lower on self-consciousness and their pattern of recall was similar for self- vs. other-referenced items. Neuroimaging studies have demonstrated that the default network regions such as medial prefrontal cortex and posterior cingulate gyrus are implicated in the processing of self-relevant social information (Gusnard et al., 2001; Martinelli et al., 2013). The default network is engaged during baseline rest periods when participants are not focused on task-directed thought and is suppressed during attention-demanding tasks (Raichle et al., 2001). Aging also alters the neural activity associated with the successful formation of memories for self-referenced information (Gutchess et al., 2010). Future studies need to directly link changes in self-understanding and performance in social problem solving and personal well-being.

## Dealing Effectively with Uncertainty

Decisions often need to be made in situations of risk and ambiguity, i.e., uncertainty. Dealing with such situations effectively is a crucial component of wisdom. In decision theory, a distinction is made between risk and ambiguity based on whether the probability associated with an outcome is known. If the objective probability of an outcome is known, it is risky; if the objective probability of an outcome is not known, it is ambiguous (Tversky and Fox, 1995). The two classic economic theories for understanding decisions under risk and ambiguity are expected utility (EU) and subjective expected utility (SEU). In EU (Morgenstern and Von Neumann, 1947), outcomes are evaluated based on their objective probabilities. In SEU (Savage, 1954), objective probabilities may not be known and outcomes are evaluated based on the decision maker’s subjective probabilities of outcomes. In both theories, decision makers multiply the probabilities and values associated with outcomes and choose the outcome that yields the greatest expected value. Attitudes towards risk and ambiguity are generally classified as “aversion” or “seeking”. Risk aversion is defined as preferring a less risky outcome to a more risky outcome with equal or greater expected value (Rabin and Thaler, 2001), e.g., choosing between $10 for sure and a 50% chance to win $20 or nothing. Conversely, risk seeking is defined as preferring a more risky outcome to a less risky outcome with equal or greater expected value. Ambiguity aversion is defined as preferring outcomes with known probabilities to outcomes with unknown probabilities; conversely, ambiguity seeking is defined preferring outcomes with unknown probabilities to outcomes with known probabilities (Epstein, 1999).

### Risk

Risky choices are usually elicited in laboratory experimental settings using a variety of behavioral tasks. These tasks can be classified as requiring decision makers to make their *decisions from* *description* or *experience* (Hertwig et al., 2004). In tasks requiring decisions from description, options are described with their outcomes and probabilities. In tasks requiring decisions from experience, these descriptions are not explicitly provided and decision makers instead rely on personal experience from making previous similar choices.

An example of a task requiring decisions from description would be choosing between $10 for sure and a 50% chance to win $20 or nothing. A large number of studies investigating risk employ this approach (Weber et al., 2004). However, this approach has been criticized on the grounds of being an unrealistic representation of typical real world situations where summary descriptions of choice outcomes and probabilities are not provided (Hertwig et al., 2004). Instead, it was argued, tasks requiring decisions from experience are more reflective of everyday life (Hertwig et al., 2004). An example of such a task would be repeatedly choosing between outcomes sampled from unknown probability distributions and this is usually done on a computer. It is worth noting that decisions from experience contain a degree of uncertainty, i.e., it is not strictly uncertain because the statistical probability can be estimated (see Rakow and Newell, 2010). Intuitively, one would expect choices to be similar on both tasks when payments are the same on average. However, this is not the case: on a decision from description task, the modal choice tends to be the risky option while the sure outcome tends to be preferred on a decision from experience task (see Hau et al., 2008; Rakow and Newell, 2010). This difference in risky choice is known as the *description-experience gap*. Several explanations for this gap have been proposed (for a review, see Hertwig and Erev, 2009).

A meta-analysis of 29 studies (*N* = 4093) using various behavioral tasks to investigate age differences in risky decision making reported mixed results (Mata et al., 2011). There was no overall significant age difference in tasks involving decisions from description although age differences varied across individual tasks. On tasks involving decisions from experience, there was a significant overall age difference: older adults were slightly more risk seeking (*d* = 0.28). However, age differences varied across individual tasks. Combining tasks involving both decisions from experience and description yielded a negligible age difference (*d* = 0.07). The authors conclude that “different tasks characteristics engender age-related differences in risky choice” (Mata et al., 2011).

### Ambiguity

An ambiguity-averse individual would rather choose an alternative where the probability distribution of the outcomes is known over one where the probabilities are unknown. Regarding uncertain outcomes, people are generally believed to display ambiguity aversion (Camerer and Weber, 1992; Keren and Gerritsen, 1999). However, there is a dearth of studies examining age differences in decision making in ambiguous situations. One study found no significant age differences in gambles (Kovalchik et al., 2005), while another found older participants to be less ambiguity-averse in gambles (Sproten et al., 2010) i.e., they were more likely to choose the ambiguous outcome to the certain outcome. The lack of studies could be due to the general difficulty in measuring pure ambiguity in laboratory experiments (Lopes, 1983). As such, more studies investigating how older people make ambiguous choices are needed.

### Intertemporal Choice

Wisdom is required to make good decisions about future life plans and goals (Baltes and Smith, 2008). Many important life decisions require making intertemporal choices, i.e., trading off time and outcomes such as money, health or happiness. For example, deciding whether to pursue higher education while earning little to no money over a few years for a potentially greater lifetime earnings in the future (Read et al., 2013). Intertemporal choices involve ambiguity since the future is uncertain (Read and Read, 2004). In experiments, participants are typically asked to choose between smaller-sooner (SS) and larger-later (LL) amounts of money, e.g., $100 today or $110 in 1 year. Standard economic theory assumes exponential, i.e., constant per period, discounting, although this is not supported by empirical evidence, which suggests that individuals tend to be impatient, i.e., they prefer SS outcomes, and discount future outcomes more (Frederick et al., 2002).

Fourteen studies examined the relationship between impatience and age.^1^ Half studies found older people to be more patient (Green et al., 1994; Harrison et al., 2002; Reimers et al., 2009; Whelan and Mchugh, 2010; Löckenhoff et al., 2011; Halfmann et al., 2013; Li et al., 2013). Four studies found no difference (Chao et al., 2009; Samanez-Larkin et al., 2011; Roalf et al., 2012; Rieger and Mata, 2015). Two studies found older people to be more impatient (Green et al., 1996; Albert and Duffy, 2012).^2^ Finally one study found a curvilinear relationship with middle-aged people the most patient, while older people were more impatient than younger people (Read and Read, 2004). The only two studies that investigated intertemporal discounting on losses both found no age effects (Löckenhoff et al., 2011; Halfmann et al., 2013).

There are several possible explanations for the discrepancies in results across studies. For example, Read and Read (2004) pointed out that an earlier study by Green et al. (1994) focussed on incomparable samples, which were quite small (*n* = 12 per group), without controlling for confounding factors such as income, etc. Similar criticisms may apply to more recent studies as well (e.g., Whelan and Mchugh, 2010; Samanez-Larkin et al., 2011; Halfmann et al., 2013). Rieger and Mata (2015) proposed another reason for their finding of no age effects in 700 Moroccans: age differences may not generalize across cultures and nationalities. Another reason pointed out by Li et al. (2013) is the difference in the average age of older participants across studies, with few studies having sufficient data for participants above 65 years old.

Finally, we noticed large differences in the questions asked across studies. For example, monetary amounts offered ranged from less than $10 to $1800 and the interval length between SS and LL varied from a few days to weeks, months and even years. Studies have found impatience to be influenced by the magnitude of the outcome, the interval length between outcomes and the delay from the present to the availability of the outcomes (e.g., Thaler, 1981; Kirby et al., 1999; Read, 2001; Read and Roelofsma, 2003). These factors may influence people of different ages differently, although no studies have examined this yet. There were also few studies investigating age differences and intertemporal choices involving losses.

### Summary

Taken together, these studies revealed that age-related changes in economic decisions involving risk, ambiguity, and intertemporal choices are determined by task characteristics, the specific age range, and a variety of other methodological factors (Frederick et al., 2002; Mata et al., 2011). There is insufficient evidence to accurately determine whether older adults make risky, ambiguous and intertemporal choices differently from younger adults.

## Concluding Remarks

Overall, the relationships between age and each of the subcomponents of wisdom remain unclear. This is a relatively young field, and it is still a challenging prospect to integrate the conflicting findings often found in this field. Several issues may contribute to the inconsistency in findings. First, there were differences in the average age across studies, with few studies having sufficient data for participants above 65 years old. For studies that used a between-subjects design, the average age for both the older and younger, i.e., comparison, group of participants could differ substantially across studies. Second, there were significant methodological variations across studies. Studies differed in the types of task used, study designs employed and in controlling for potential confounding factors. Third, a number of studies, especially in the neurosciences, used small sample sizes, which undermines the reliability of the findings due to a lack of statistical power (Button et al., 2013). These issues have been consistently documented in the aging literature (Rhodes, 1983; Kite and Johnson, 1988; Thornton and Dumke, 2005; Ng and Feldman, 2008).

We also uncovered several gaps in the literature that future research can address. First, participants in most of the studies were mainly from Anglo countries. This is consistent with findings from Henrich et al. (2010) who questioned generalizability of behavioral science findings across human populations due to the overwhelming number of studies on student samples from Western, Educated, Industrialized, Rich and Democratic countries, and in particular USA. The need for non-Anglo samples is relevant given the finding of age-related differences in social wisdom between Japanese and American adults (Grossmann et al., 2012). Second, there is a lack of research into how factors such as gender, individual differences in personality, culture and the environment influence wisdom in the elderly. Third, studies typically employed cross-sectional and not longitudinal designs, which is in line with other fields examining age differences (Rhodes, 1983; Ng and Feldman, 2008). This results in cohort effects (Rhodes, 1983), which threaten the internal validity of studies and limit our understanding of age-related changes in the subcomponents of wisdom over the lifetime.

There is a compelling need for future studies to address the aforementioned issues in order to better understand how older people make economic and social decisions. This is especially important given the impending demographic shift to an older society. The prejudice and stereotypes may limit meaningful participation in society, e.g., jobs, and affect the mental health states of old people, who may even internalize and play into the stereotypes in self-fulfilling prophecies (Taylor and Walker, 1994, 1998; Levy, 2001; Coudin and Alexopoulos, 2010). A better understanding of the actual changes in old age can help foster a more inclusive society that taps on the expertise and skills of those older. This in turn may help alleviate feelings of social isolation, loneliness and depression (Perlman and Peplau, 1981). Understanding these issues is pertinent to implementing early interventions aimed at preventing a wide range of mental health problems, and has broad implications for social policies aimed at the elderly.

## Conflict of Interest Statement

The authors declare that the research was conducted in the absence of any commercial or financial relationships that could be construed as a potential conflict of interest.

## Footnotes

^1^Studies either measure the discount rate or impatience but holding the amounts and time constant, a higher discount rate implies greater impatience.

^2^To be precise, Green et al. (1996) found lower income adults more impatient than upper income younger adults but no differences between upper income younger and older adults.
